# RAMESES II reporting standards for realist evaluations

**DOI:** 10.1186/s12916-016-0643-1

**Published:** 2016-06-24

**Authors:** Geoff Wong, Gill Westhorp, Ana Manzano, Joanne Greenhalgh, Justin Jagosh, Trish Greenhalgh

**Affiliations:** Nuffield Department of Primary Care Health Sciences, University of Oxford, Radcliffe Primary Care Building, Radcliffe Observatory Quarter, Woodstock Road, Oxford, OX2 6GG UK; Community Matters, PO Box 443, Mount Torrens, South Australia 5244 Australia; School of Sociology and Social Policy, University of Leeds, Leeds, LS2 9JT UK; Centre for Advancement in Realist Evaluation and Synthesis, Institute of Psychology, Health and Society, University of Liverpool, Waterhouse Building, Block B, Brownlow Street, Liverpool, L69 3GL UK

**Keywords:** Realist evaluation, Delphi approach, Reporting guidelines

## Abstract

**Background:**

Realist evaluation is increasingly used in health services and other fields of research and evaluation. No previous standards exist for reporting realist evaluations. This standard was developed as part of the RAMESES II project. The project’s aim is to produce initial reporting standards for realist evaluations.

**Methods:**

We purposively recruited a maximum variety sample of an international group of experts in realist evaluation to our online Delphi panel. Panel members came from a variety of disciplines, sectors and policy fields. We prepared the briefing materials for our Delphi panel by summarising the most recent literature on realist evaluations to identify how and why rigour had been demonstrated and where gaps in expertise and rigour were evident. We also drew on our collective experience as realist evaluators, in training and supporting realist evaluations, and on the RAMESES email list to help us develop the briefing materials.

Through discussion within the project team, we developed a list of issues related to quality that needed to be addressed when carrying out realist evaluations. These were then shared with the panel members and their feedback was sought. Once the panel members had provided their feedback on our briefing materials, we constructed a set of items for potential inclusion in the reporting standards and circulated these online to panel members. Panel members were asked to rank each potential item twice on a 7-point Likert scale, once for relevance and once for validity. They were also encouraged to provide free text comments.

**Results:**

We recruited 35 panel members from 27 organisations across six countries from nine different disciplines. Within three rounds our Delphi panel was able to reach consensus on 20 items that should be included in the reporting standards for realist evaluations. The overall response rates for all items for rounds 1, 2 and 3 were 94 %, 76 % and 80 %, respectively.

**Conclusion:**

These reporting standards for realist evaluations have been developed by drawing on a range of sources. We hope that these standards will lead to greater consistency and rigour of reporting and make realist evaluation reports more accessible, usable and helpful to different stakeholders.

## Background

Realist evaluation is a form of theory-driven evaluation, based on a realist philosophy of science [1, 2] that addresses the questions, ‘what works, for whom, under what circumstances, and how’. The increased use of realist evaluation in the assessment of complex interventions [3] is due to the realisation by many evaluators and commissioners that coming up with solutions to complex problems is challenging and requires deeper insights into the nature of programmes and implementation contexts. These problems have multiple causes operating at both individual and societal levels, and the interventions or programmes designed to tackle such problems are themselves complex. They often have multiple, interconnected components delivered individually or targeted at communities or populations, with success dependent both on individuals’ responses and on the wider context. What works for one family, or organisation, or city may not work in another. Effective complex interventions or programmes are difficult to design and evaluate. Effective evaluations need to be able to consider how, why, for whom, to what extent, and in what context complex interventions work. Realist evaluations can address these challenges and have indeed addressed numerous topics of central relevance in health services research [4–6] and other fields [7, 8].

### What is realist evaluation?

The methodology of realist evaluation was originally developed in the 1990’s by Pawson and Tilley to address the question ‘what works, for whom, in what circumstances, and how?’ in a broad range of interventions [1]. Realist evaluations, in contrast to other forms of theory driven evaluations [9], must be underpinned by a realist philosophy of science. In realist evaluations it is assumed that social systems and structures are ‘real’ (because they have real effects) and also that human actors respond differently to interventions in different circumstances. To understand how an intervention might generate different outcomes in different circumstances, a realist evaluation examines how different programme mechanisms, namely underlying changes in the reasoning and behaviour of participants, are triggered in particular contexts. Thus, programmes are believed to ‘work’ in different ways for different people in different situations [10]:

- Social programmes (or interventions) attempt to create change by offering (or taking away) resources to participants or by changing contexts within which decisions are made (for example, changing laws or regulations);
- Programmes ‘work’ by enabling or motivating participants to make different choices;
- Making and sustaining different choices requires a change in a participant’s reasoning and/or the resources available to them;
- The contexts in which programmes operate make a difference to and thus shape the mechanisms through which they work and thus the outcomes they achieve;
- Some factors in the context may enable particular mechanisms to operate or prevent them from operating;
- There is always an interaction between context and mechanism, and that interaction is what creates the programme’s impacts or outcomes (Context + Mechanism = Outcome);
- Since programmes work differently in different contexts and through different mechanisms, programmes cannot simply be replicated from one context to another and automatically achieve the same outcomes. Theory-based understandings about ‘what works, for whom, in what contexts, and how’ are, however, transferable;
- One of the tasks of evaluation is to learn more about ‘what works for whom’, ‘in which contexts particular programmes do and don’t work’ and ‘what mechanisms are triggered by what programmes in what contexts’.

In a realist evaluation the assumption is that programmes are ‘theories incarnate’ [1]. That is, whenever a programme is designed and implemented, it is underpinned by one or more theories about what ‘might cause change’, even though that theory or theories may not be explicit. Undertaking a realist evaluation requires that the theories within a programme are made explicit, by developing clear hypotheses about how, and for whom, to what extent, and in what contexts a programme might ‘work’. The evaluation of the programme tests and refines those hypotheses. The data collected in a realist evaluation needs to enable such testing and so should include collecting data about: programme impacts and the processes of programme implementation, the specific aspects of programme context that might impact on programme outcomes, and how these contexts shape the specific mechanisms that might be creating change. This understanding of how a particular aspects of the context shapes the mechanism which leads to outcomes can be expressed as a context-mechanism-outcome (CMO) configuration and is the analytical unit on which realist evaluation is built. Eliciting, refining and testing CMO configurations allows a deeper and more detailed understanding of for whom, in what circumstances and why the programme works. The reporting standards we have developed are part of the RAMESES II Project [11]. In addition, we are developing training materials to support realist evaluators and these will be made freely available on: www.ramesesproject.org.

A realist approach has particular implications for the design of an evaluation and the roles of participants. For example, rather than comparing the outcomes for participants who have and have not taken part in a programme (as is done in a randomised controlled or quasi-experimental designs), a realist evaluation compares CMO configurations within programmes or across sites of implementation. It asks, for example, whether a programme works more or less well in different localities (and if so, how and why), or for different participants. These participants may be programme designers, implementers and recipients.

When seeking input from participants, it is assumed that different participants have different perspectives, information and understandings about how programmes are supposed to work and whether they in fact do. As such, data collection processes (interviews, focus groups, questionnaires and so on) need to be constructed so that they are able to identify the particular information that those stakeholder groups will have in a realist way [12, 13]. These data may then be used to confirm, refute or refine theories about the programme. More in depth discussions about the philosophical underpinnings of realist evaluation and its origins may be found in the training materials we are developing and other sources [1, 2, 11].

### Why are reporting standards needed?

Within health services research, reporting standards are common and increasingly expected (for example, [14–16]). Within realist research, reporting standards have already been developed for realist syntheses (also known as reviews) [17, 18]. The rationale behind our reporting standards is that they will guide evaluators about what needs to be reported in a realist evaluation. This should serve two purposes. It will help readers to understand the programme under evaluation itself and the findings from the evaluation and it will provide readers with relevant and necessary information to enable them to assess the quality and rigour of the evaluation.

Such reporting guidance is important for realist evaluations. Since its development almost two decades ago, realist evaluation has gained increasing interest and application in many fields of research, including health services research. Published literature [19, 20], postings in the RAMESES email list we have run since 2011 [21], and our experience as trainers and mentors in realist methodologies all suggest that there is confusion and misunderstanding among evaluators, researchers, journal editors, peer reviewers and funders about what counts as a high quality realist evaluation and what, conversely, as substandard. Even though experts still differ on detailed conceptual methodological issues, the increasing popularity of realist evaluation has prompted us to develop baseline reporting (and later) quality standards which, we anticipate, will advance the application of realist theory and methodology. We anticipate that both the reporting standards and the quality standards will themselves evolve over time, as the theory and methodology evolve.

The present study aims to produce initial reporting standards for realist evaluations.

## Methods

The methods we used to develop these reporting standards have been published elsewhere [11]. In summary, we purposively recruited an international group of experts to our online Delphi panel. We aimed to achieve maximum variety and sought out panel members who use realist evaluation in a variety of disciplines, sectors and policy fields. To prepare the briefing materials for our Delphi panel, we collated and summarised the most recent literature on realist evaluations, seeking to identify how and why rigour had been demonstrated and where gaps in expertise and rigour were evident. We also drew on our collective experience as realist evaluators, in training and supporting realist evaluations and on the RAMESES email list to help us develop the briefing materials.

Through discussion within the project team, we considered the findings of our review of recent examples of realist evaluations and developed a list of issues related to quality that needed to be addressed when carrying out realist evaluations. These were then shared with the panel members and additional issues were sought. Once the panel members had provided their feedback on our briefing materials we constructed a set of items for potential inclusion in the reporting standards and circulated these online to panel members. Panel members were asked to rank each potential item twice on a 7-point Likert scale (1 = strongly disagree to 7 = strongly agree), once for relevance (i.e. should an item on this theme/topic be included at all in the standards?) and once for validity (i.e. to what extent do you agree with this item as currently worded?). They were also encouraged to provide free text comments. We ran the Delphi panel over three rounds between June 2015 and January 2016.

### Description of panel and items

We recruited 35 panel members from 27 organisations across six countries. They comprised of evaluators of health services (23), public policy (9), nursing (6), criminal justice (6), international development (2), contract evaluators (3), policy and decision makers (2), funders of evaluations (2) and publishing (2) (note that some individuals had more than one role).

In round 1 of the Delphi panel, 33 members provided suggestions for items that should be included in the reporting standards and/or comments on the nature of the standards themselves. In rounds 2 and 3, panel members ranked the items for relevance and validity. For round 2, the panel was presented with 22 items to rank. The overall response rate across all items for this round was 76 %. Based on the rankings and free text comments our analysis indicated that two items needed to be merged and one item removed. Minor revisions were made to the text of the other items based on the rankings and free text comments. After discussion within the project team we judged that only one item (the newly created merged item) needed to be returned to round 3 of the Delphi panel. The response rate for round 3 was 80 %. Consensus was reached within three rounds on both the content and wording of a 20 item reporting standard. Table 1 provides an overview of these items. Those using the list of Items in Table 1 to help guide the reporting of their realist evaluations may find the last two columns (‘Reported in document’ and ‘Page(s) in document’) as a useful way to indicate to others where in the document each item has been reported.

**Table 1:** List of items to be included when reporting realist evaluations

TITLE			Reported in document Y/N/Unclear	Page(s) in document
1		In the title, identify the document as a realist evaluation		
SUMMARY OR ABSTRACT				
2		Journal articles will usually require an abstract, while reports and other forms of publication will usually benefit from a short summary. The abstract or summary should include brief details on: the policy, programme or initiative under evaluation; programme setting; purpose of the evaluation; evaluation question(s) and/or objective(s); evaluation strategy; data collection, documentation and analysis methods; key findings and conclusions Where journals require it and the nature of the study is appropriate, brief details of respondents to the evaluation and recruitment and sampling processes may also be included Sufficient detail should be provided to identify that a realist approach was used and that realist programme theory was developed and/or refined		
INTRODUCTION				
3	Rationale for evaluation	Explain the purpose of the evaluation and the implications for its focus and design		
4	Programme theory	Describe the initial programme theory (or theories) that underpin the programme, policy or initiative		
5	Evaluation questions, objectives and focus	State the evaluation question(s) and specify the objectives for the evaluation. Describe whether and how the programme theory was used to define the scope and focus of the evaluation		
6	Ethical approval	State whether the realist evaluation required and has gained ethical approval from the relevant authorities, providing details as appropriate. If ethical approval was deemed unnecessary, explain why		
METHODS				
7	Rationale for using realist evaluation	Explain why a realist evaluation approach was chosen and (if relevant) adapted		
8	Environment surrounding the evaluation	Describe the environment in which the evaluation took place		
9	Describe the programme policy, initiative or product evaluated	Provide relevant details on the programme, policy or initiative evaluated		
10	Describe and justify the evaluation design	A description and justification of the evaluation design (i.e. the account of what was planned, done and why) should be included, at least in summary form or as an appendix, in the document which presents the main findings. If this is not done, the omission should be justified and a reference or link to the evaluation design given. It may also be useful to publish or make freely available (e.g. online on a website) any original evaluation design document or protocol, where they exist		
11	Data collection methods	Describe and justify the data collection methods – which ones were used, why and how they fed into developing, supporting, refuting or refining programme theory Provide details of the steps taken to enhance the trustworthiness of data collection and documentation		
12	Recruitment process and sampling strategy	Describe how respondents to the evaluation were recruited or engaged and how the sample contributed to the development, support, refutation or refinement of programme theory		
13	Data analysis	Describe in detail how data were analysed. This section should include information on the constructs that were identified, the process of analysis, how the programme theory was further developed, supported, refuted and refined, and (where relevant) how analysis changed as the evaluation unfolded		
RESULTS				
14	Details of participants	Report (if applicable) who took part in the evaluation, the details of the data they provided and how the data was used to develop, support, refute or refine programme theory		
15	Main findings	Present the key findings, linking them to contexts, mechanisms and outcome configurations. Show how they were used to further develop, test or refine the programme theory		
DISCUSSION				
16	Summary of findings	Summarise the main findings with attention to the evaluation questions, purpose of the evaluation, programme theory and intended audience		
17	Strengths, limitations and future directions	Discuss both the strengths of the evaluation and its limitations. These should include (but need not be limited to): (1) consideration of all the steps in the evaluation processes; and (2) comment on the adequacy, trustworthiness and value of the explanatory insights which emerged In many evaluations, there will be an expectation to provide guidance on future directions for the programme, policy or initiative, its implementation and/or design. The particular implications arising from the realist nature of the findings should be reflected in these discussions		
18	Comparison with existing literature	Where appropriate, compare and contrast the evaluation’s findings with the existing literature on similar programmes, policies or initiatives		
19	Conclusion and recommendations	List the main conclusions that are justified by the analyses of the data. If appropriate, offer recommendations consistent with a realist approach		
20	Funding and conflict of interest	State the funding source (if any) for the evaluation, the role played by the funder (if any) and any conflicts of interests of the evaluators		

### Scope of the reporting standards

These reporting standards are intended to help evaluators, researchers, authors, journal editors, and policy- and decision-makers to know and understand what should be reported when writing up a realist evaluation. They are not intended to provide detailed guidance on how to conduct a realist evaluation; for this, we would suggest that interested readers access summary articles or publications on methods [1, 19, 20, 22, 23]. These reporting standards apply only to realist evaluation. A list of publication or reporting guidelines for other evaluation methods can be found on the EQUATOR Network’s website [24], but at present none of these relate specifically to realist evaluations. As part of the RAMESES II project we are also developing quality standards which will be available as a separate publication and training materials for realist evaluations [11].

### How to use these reporting standards

The layout of this document is based on the RAMESES publication standards: realist syntheses [17, 18], which itself was based on previous methodological publications (in particular, on the ‘Explanations and Elaborations’ document of the PRISMA statement [25]. After each item there is an exemplar drawn from publically available evaluations followed by a rationale for its inclusion. Within these standards, we have drawn our exemplar texts mainly from realist evaluations that have been published in peer review journals, as these were easy to access and publically available. Our choice of exemplar texts should not be taken to imply that the standard of reporting of realist evaluations that have not been published in peer review journals is in any way substandard.

The exemplar text is provided to illustrate how an item might be written up in a report. However, each exemplar has been extracted out of a larger document and so important contextual information has been omitted. It may thus be necessary to consult the original document from which the exemplar text was drawn to fully understand the evaluation it refers to.

What might be expected for each item has been set out within these reporting standards, but authors will still need to exercise judgement about how much information to include. The information reported should be sufficient to enable readers to judge that a realist evaluation has been planned, executed, analysed and reported in a coherent, trustworthy and plausible fashion, both against the guidance set out within an item and for the overall purposes of the evaluation itself.

Evaluations are carried out for different purposes and audiences. Realist evaluations in the academic literature are usually framed as research, but many realist evaluations in the grey literature were commissioned as evaluations, not research. Hence, the items listed in Table 1 should be interpreted flexibly depending on the purpose of the evaluation and the needs of the audience. This means that not all evaluation reports need necessarily be reported in an identical way. It would be reasonable to expect that the order in which items are reported may vary and not all items will be required for every type of evaluation report. As a general rule, if an item within these reporting standards has been excluded from the write-up of a realist evaluation, a justification should be provided.

### The RAMESES reporting standards for realist evaluations

#### Item 1: Title

In the title, identify the document as a realist evaluation.

#### Example

*How do you modernize a health service? A realist evaluation of whole-scale transformation in London *[26].

#### Explanation

Our background searching has shown that some realist evaluations are not flagged as such in the title and may also be inconsistently indexed, and hence are more difficult to locate. Realist evaluation is a specific theoretical and methodological approach, and should be carefully distinguished from evaluations that use different approaches (e.g. such as other theory-based approaches) [27]. Researchers, policy staff, decision-makers and other knowledge users may wish to be able to locate reports using realist approaches. Adding the term ‘realist evaluation’ as a keyword may also aid searching and identification.

#### Item 2: Summary or abstract

Journal articles will usually require an abstract while reports and other forms of publication will usually benefit from a short summary. The abstract or summary should include brief details on the policy, programme or initiative under evaluation; programme setting; purpose of the evaluation; evaluation question(s) and/or objective(s); evaluation strategy; data collection, documentation and analysis methods; key findings and conclusions.

Where journals require it and the nature of the study is appropriate, brief details of respondents to the evaluation and recruitment and sampling processes may also be included.

Sufficient detail should be provided to identify that a realist approach was used and that realist programme theory was developed and/or refined.

#### Example

*The current project conducted an evaluation of a community-based addiction program in Ontario, Canada, using a realist approach. Client-targeted focus groups and staff questionnaires were conducted to develop preliminary theories regarding how, for whom, and under what circumstances the program helps or does not help clients. Individual interviews were then conducted with clients and caseworkers to refine these theories. Psychological mechanisms through which clients achieved their goals were related to client needs, trust, cultural beliefs, willingness, self-awareness and self-efficacy. Client, staff and setting characteristics were found to affect the development of mechanisms and outcomes* [28].

#### Explanation

Evaluators will need to provide either a summary or abstract depending on the type of document they wish to produce. Abstracts are often needed for formal academic publications and summaries for project reports.

Apart from the title, a summary or abstract is often the only source of information accessible to searchers unless the full text document is obtained. Many busy knowledge users will often not have the time to read an entire evaluation report or publication in order to determine its relevance and initially only access the summary or abstract. The information in it must allow the reader to decide whether the evaluation is a realist evaluation and relevant to their needs.

The brief summary we refer to here does not replace the Executive Summary that many evaluation reports will provide. In a realist evaluation, the Executive Summary should include concise information about programme theory and CMO configurations, as well as the other items described above.

### Introduction section

#### Item 3: Rationale for evaluation

Explain the purpose of the evaluation and the implications for its focus and design.

#### Example

*In this paper, we aim to demonstrate how the realist evaluation approach helps in advancing complex systems thinking in healthcare evaluation. We do this by comparing the outcomes of cases which received a capacity-building intervention for health managers and explore how individual, institutional and contextual factors interact and contribute to the observed outcomes * [29].

#### Explanation

Realist evaluations are used for many types of programmes, projects, policies and initiatives (all referred to here as ‘programmes’ or ‘evaluands’ – ‘that which is evaluated’ – for ease of reference). They are also conducted for multiple purposes (e.g. to develop or improve design and planning; understand whether and how new programmes work; improve effectiveness overall or for particular populations; improve efficiency; inform decisions about scaling programmes out to other contexts; or to understand what is causing variations in implementation or outcomes). The purpose has significant implications for the focus of the work, the nature of questions, the design, and the choice of methods and analysis [30]. These issues should be clearly described in the ‘background’ section of a journal article or the introduction to an evaluation report. Where relevant, this should also describe what is already known about the subject matter and the ‘knowledge gaps’ that the evaluation sought to fill.

#### Item 4: Programme theory

Describe the initial programme theory (or theories) that underpin the programme, policy or initiative.

#### Example

*The programme theory (highlighting the underlying psychosocial mechanisms providing the active ingredients to facilitate change), was:*

- *By facilitating critical discussion of ‘what works’ (academic and practice-based evidence, in written and verbal form), across academic and field experts and amongst peers, understanding of the practical application and potential benefits of evidence use would be increased, uptake of the evidence would be facilitated and changes to practice would follow.*

*A secondary programme theory was that:*

- *Allowing policy, practice and academic partners to come together to consider their common interests, share the challenges and opportunities of working on public health issues, trusting relationship would be initiated and these would be followed-up by future contact and collaborative work* [31].

#### Explanation

Realist evaluations set out to develop, support, refute or refine aspects of realist programme theory (or theories). All programmes or initiatives will (implicitly or explicitly) have a programme theory or theories [32] – ideas about how the programme is expected to cause its intended outcomes – and these should be articulated here. Initial programme theories may or may not be realist in nature. As an evaluation progresses, a programme theory that was not initially realist in nature will need to be developed and refined so that it becomes a realist programme theory (that is, addressing all of context, mechanism and outcome) [33].

Programmes are theories incarnate. Within a realist evaluation, programme theory can serve many functions. One of its functions is to describe and explain (some of) how and why, in the ‘real world’, a programme ‘works’, for whom, to what extent and in which contexts (the assumption that the programmes will only work in some contexts and for some people, and that it may fire different mechanisms in different circumstances, thus generating different outcomes, is one of the factors that distinguishes realist programme theory from other types of programme theory). Other functions include focusing an evaluation, identifying questions, and determining what types of data need to be collected and from whom and where, in order to best support, refute or refine the programme theory. The refined programme theory can then serve many purposes for evaluation commissioners and end users [34]. It may support planning and decision-making about programme refinement, scaling out and so on.

Different processes can be used for ‘uncovering’ or developing initial programme theory, including literature review, programme documentation review, and interviews and/or focus groups with key informants. The processes used to develop the programme theory are usually different from those used later to refine it. The programme theory development processes needs to be clearly reported for the sake of transparency. The processes used for programme theory development may be reported here or in ‘Item 13: Data analysis’.

A figure or diagram may aid in the description of the programme theory. It may be presented early in the report or as an appendix.

Sometimes, the focus of the evaluation will not be ‘the programme itself’ but a particular aspect of, or question about, a programme (for example, how the programme affects other aspects of the system in which it operates). The theory that is developed or used in any realist evaluation will be relevant to the particular questions under investigation.

#### Item 5: Evaluation questions, objectives and focus

State the evaluation question(s) and specify the objectives for the evaluation. Describe whether and how the programme theory was used to define the scope and focus of the evaluation.

#### Example

*The objective of the study was to analyse the management approach at CRH [Central Regional Hospital]. We formulated the following research questions: (1) What is the management team's vision on its role? (2) Which management practices are being carried out? (3) What is the organisational climate? … ; (4) What are the results? (5) What are the underlying mechanisms explaining the effect of the management practices?* [6].

#### Explanation

Realist evaluation questions contain some or all of the elements of ‘what works, how, why, for whom, to what extent and in what circumstances, in what respect?’ Specifically, realist evaluation questions need to reflect the underlying purpose of realist evaluation – that is to explain (how and why) rather than only describe outcome patterns.

Note that the term ‘outcome’ will mean different things in different kinds of evaluations. For example, it may refer to ‘patterns of implementation’ in process evaluations; ‘patterns of efficiency or cost effectiveness for different populations’ in economic evaluation; as well as outcomes and impacts in the normal uses of the term. Moreover, realist evaluation questions may deal with other traditional evaluation questions of value, relevance, effectiveness, efficiency, and sustainability, as well as questions of ‘outcomes’ [35]. However, they will apply the same kinds of question structure to those issues (for example, effectiveness for whom, how and why; efficiency in what circumstances, how and why, and so on).

Because a particular evaluation will never be able to address all potential questions or issues, the scope of the evaluation has to be clarified. This may involve discussion and negotiation with (for example) commissioners of the evaluation, context experts, research funders and/or users. The processes used to establish purposes, scope, questions, and/or objectives should be described. Whether and how the programme theory was used in determining the scope of the evaluation should be clearly articulated.

In the real world, the programme being evaluated does not sit in a vacuum. Instead, it is thrust into a messy world of pre-existing programmes, a complex policy environment, multiple stakeholders and so on [2, 36]. All of these may have a bearing on (for example) the research questions, focus and constraints of the evaluation. Reporting should provide information to the reader about the policy and other circumstances that may have influenced the purposes, scope, questions and/or objectives of the evaluation.

Given the iterative nature of realist evaluation, if the purposes, scope, questions, objectives, programme theory and/or protocol changed over the course of the evaluation, it should either be reported here or in ‘Item 15: Main findings’.

#### Item 6: Ethical approval

State whether the realist evaluation required and has gained ethical approval from the relevant authorities, providing details as appropriate. If ethical approval was deemed unnecessary, explain why.

#### Example

*The study was reported to the Danish Data Protection Agency (2008-41-2322), the Ethics Committee of the Capital Region of Denmark (REC; reference number 0903054, document number 230436) and Trials Registration (ISTCTN54243636) and performed in accordance with the ethical recommendations of the Helsinki Declaration *[37].

#### Explanation

Realist evaluation is a form of primary research and will usually involve human participants. It is important that evaluations are conducted ethically. Evaluators come from a range of different professional backgrounds and work in diverse fields. This means that different professional ethical standards and local ethics regulatory requirements will apply. Evaluators should ensure that they aware of and comply with their professional obligations and local ethics requirements throughout the evaluation.

Specifically, one challenge that realist evaluations may face is that legitimate changes may be required to the methods used and participants recruited as the evaluation evolves. Anticipating that such changes may be needed is important when seeking ethical approval. Flexibility may need to be built into the project to allow for updating ethics approvals and be explained to those who provide ethics approvals.

### Methods section

#### Item 7: Rationale for using realist evaluation

Explain why a realist evaluation approach was chosen and (if relevant) adapted.

#### Example

*This study used realist evaluation methodology… A central tenet of realist methodology is that programs work differently in different contexts–hence a community partnership that achieves ‘success’ in one setting may ‘fail’ (or only partially succeed) in another setting, because the mechanisms needed for success are triggered to different degrees in different contexts. A second tenet is that for social programs, mechanisms are the cognitive or affective responses of participants to resources offered [Reference]. Thus the realist methodology is well suited to the study of CBPR [Community-Based Participatory Research], which can be understood, from an ecological perspective, as a multiple intervention strategies implemented in diverse community contexts [References] dependant on the dynamics of relationships among all stakeholders *[38].

#### Explanation

Realist evaluation is firmly rooted in a realist philosophy of science. It places particular emphasis on understanding causation (in this case, understanding how programmes generate outcomes) and how causal mechanisms are shaped and constrained by social, political, economic (and so on) contexts. This makes it particularly suitable for evaluations of certain topics and questions – for example, complex social programmes that involve human decisions and actions [1]. It also makes realist evaluation less suitable than other evaluation approaches for certain topics and questions – for example those which seek primarily to determine the average effect size of a simpler intervention administered in a limited range of conditions [39]. The intent of this item in the reporting standard is not that the philosophical principles should be described in every evaluation but that the relevance of the approach to the topic should be made explicit.

Published evaluations demonstrate that some evaluators have deliberately adapted or been ‘inspired’ by the realist evaluation approach as first described by Pawson and Tilley. The description and rationale for any adaptations made or what aspects of the evaluations have been ‘inspired’ by realist evaluation should be provided. Where evaluation approaches have been combined, this should be described and the implications for methods made explicit. Such information will allow criticism and debate amongst users and evaluators on suitability of those adaptations for the particular purposes of the evaluation.

#### Item 8: Environment surrounding the evaluation

Describe the environment in which the evaluation took place.

#### Example

*The … SMART [Self-Management Supported by Assistive, Rehabilitation and Telecare Technologies] Rehabilitation research programme*(*www.thesmartconsortium.org*) *[Reference], began in 2003 to develop and test a prototype telerehabilitation device (The SMART Rehabilitation Technology System) for therapeutically prescribed stroke rehabilitation for the upper limb. The aim was to enable the user to adopt theories and principles underpinning post-stroke rehabilitation and self-management. This included the development and initial testing of the SMART Rehabilitation Technology System (SMART 1) [References] and then from 2007, a Personalised Self-Management System for chronic disease (SMART 2) [References]* [40].

#### Explanation

Explain and describe the environment in which the programme was evaluated. This may (for example) include details about the locations, policy landscape, stakeholders, service configuration and availability, funding, time period and so on. Such information enables the reader to make sense of significant factors affecting the evaluand at differing levels (e.g. micro, meso and macro) and time points. This item may be reported here or earlier (e.g. in the Introduction section).

This description does not substitute for ‘context’ in realist explanation, which refers to the specific aspects of context which affect how specific mechanisms operate (or do not). Rather, this overall description serves to orient the reader to the evaluand and the evaluation in general.

#### Item 9: Describe the programme, policy, initiative or product evaluated

Provide relevant details on the programme, policy or initiative evaluated.

#### Example

*In a semirural locality in the North East of England, 14 general practitioner *(*GP*) *practices covering a population of 78,000 implemented an integrated care pathway *(*ICP*) *in order to improve palliative and end-of-life care in 2009/2010. The ICP is still in place and is coordinated by a multidisciplinary, multi-organisational steering group with service user involvement. It is delivered in line with national strategies on advance care planning *(*ACP*) *and end-of-life care [References] and aims to provide high-quality care for all conditions regardless of diagnosis. It requires each GP practice to develop an accurate electronic register of patients through team discussion using an agreed palliative care code, emphasising the importance of early identification and registration of those with any life-limiting illness. This enables registered patients to have access to a range of interventions such as ACP, anticipatory medication and the Liverpool Care Pathway for the Dying Patient *(*LCP*) [*Reference*]. *In order to register patients, health care professionals use the ‘surprise question’ which aims to identify patients approaching the last year of their life [Reference]. The register is a tool for the management of primary care patients; it does not involve family and patient notification. Practice teams then use a palliative rather than a curative ethos in consultation by determining patients’ future wishes*. *Descriptive GP practice data analysis had indicated that palliative care registrations had increased since the implementation of the ICP but more detailed analysis was required* [41].

#### Explanation

Realist evaluation may be used in a wide range of sectors (e.g. health, education, natural resource management, education, climate change), by a wide range of evaluators (academics, consultants, in-house evaluators, content experts) and on diverse evaluands (programmes, policies, initiatives and products), focusing on different stages of their implementation (development, pilot, process, outcome or impact) and considering outcomes of different types (quality, effectiveness, efficiency, sustainability) for different levels (whole systems, organisations, providers, end participants).

It should not be assumed that the reader will be familiar with the nature of the evaluand. The evaluand should be adequately described: what does it consist of, who does it target, who provides it, over what geographical reach, what is it supposed to achieve, and so on. Where the evaluation focuses on a specific aspect of the evaluand (e.g. a particular point in its implementation chain, a particular hypothesised mechanism, a particular effect on its environment), this should be identified – either here or in ‘Item 5: Evaluation questions, objectives and focus’ above.

#### Item 10: Describe and justify the evaluation design

A description and justification of the evaluation design (i.e. the account of what was planned, done and why) should be included, at least in summary form or as an appendix, in the document which presents the main findings. If this is not done, the omission should be justified and a reference or link to the evaluation design given. It may also be useful to publish or make freely available (e.g. online on a website) any original evaluation design document or protocol, where they exist.

#### Example

*An example of a detailed realist evaluation study protocol is: Implementing health research through academic and clinical partnerships: a realistic evaluation of the Collaborations for Leadership in Applied Health Research and Care (CLAHRC)* [42].

#### Explanation

The design for a realist evaluation may differ significantly from some other evaluation approaches. The methods, analytic techniques and so on will follow from the purpose, evaluation questions and focus. Further, as noted in Item 5 above, the evaluation question(s), scope and design may evolve over the course of the evaluation [19]. An accessible summary of what was planned in the protocol or evaluation design, in what order, and why it is useful for interpreting the evaluation. Where an evaluation design changes over time, description of the nature of and rationale for the changes can aid transparency.

Sometimes evaluations can involve a large number of steps and processes. Providing a diagram or figure of the overall structure of the evaluation may help to orient the reader [43].

Significant factors affecting the design (e.g. time or funding constraints) may also usefully be described.

#### Item 11: Data collection methods

Describe and justify the data collection methods – which ones were used, why and how they fed into developing, supporting, refuting or refining programme theory.

Provide details of the steps taken to enhance the trustworthiness of data collection and documentation.

#### Example

*An important tenet of realist evaluation is making explicit the assumptions of the programme developers. Over two dozen local stakeholders attended three ‘hypothesis generation’ workshops before fieldwork started, where many CMO configurations were proposed. These were then tested through data collection of observations, interviews and documentation.*

*Fifteen formal observations of all Delivering Choice services occurred at two time points: 1) baseline (August 2011) and 2) mid-study (Nov–Dec 2011). These consisted of a researcher sitting in on training sessions facilitated by the End of Life Care facilitators with care home staff and shadowing Delivering Choice staff on shifts at the Out of Hours advice line, Discharge in Reach service and coordination centres. Researchers also accompanied the personal care team on home visits on two occasions. Notes were taken while conducting formal observations and these were typed up and fed into the analysis *[44].

#### Explanation

Because of the nature of realist evaluation, a broad range of data may be required and a range of methods may be necessary to collect them. Data will be required for all of CMOs. Data collection methods should be adequate to capture intended and unintended outcomes, and the context-mechanism interactions that generated them. Data about outcomes should be obtained. Where possible, data about outcomes should be triangulated (at least using different sources, if not different types, of information) [20].

Commonly, realist evaluations use more than one data method to gather data. Administrative and monitoring data for the programme or policy, existing data sets (e.g. census data, health systems data), field notes, photographs, videos or sound recordings, as well as data collected specifically for the evaluation may all be required. The only constraints are that the data should allow analysis of contexts, mechanisms and outcomes relevant to the programme theory and to the purposes of and the questions for the evaluation.

Data collection tools and processes may need to be adapted to suit realist evaluation. The specific techniques used or adaptations made to instruments or processes should be described in detail. Judgements can then be made on whether the approaches chosen, instruments used and adaptations made are capable of capturing the necessary data, in formats that will be suitable for realist analysis [45].

For example, if interviews are used, the nature of the data collected must change from only accessing respondents’ interpretations of events, or ‘meanings’ (as is often done in constructivist approaches) to identifying causal processes (i.e. mechanisms) or relevant elements of context – which may or may not have anything to do with respondents’ interpretations [13].

Methods for collecting and documenting data (for example, translation and transcription of qualitative data; choices between video or oral recording; and the structuring of quantitative data systems) are all theory driven. The rationale for the methods used and their implications for data analysis should be explained.

It is important that it is possible to judge whether the processes used to collect and document the data used in a realist evaluation are rational and applied consistently. For example, a realist evaluation might report that all data from interviews were audio taped and transcribed verbatim and numerical data were entered into a spreadsheet, or collected using particular software.

#### Item 12: Recruitment process and sampling strategy

Describe how respondents to the evaluation were recruited or engaged and how the sample contributed to the development, support, refutation or refinement of programme theory.

#### Example

*To develop an understanding of how the ISP [Interpersonal Skills Profile] was actually used in practice, three groups were approached for interviews, including practitioners from each field of practice. Practice education facilitators (PEFs) –whose role was to support all health care professionals involved with teaching and assessing students in the practical setting and who had wide experience of supporting mentors – were approached to gain a broad overview. Education champions (ECs) – who were lecturers responsible for supporting mentors and students in particular areas, like a hospital or area in the community – provided an HEI perspective. Mentors were invited to contribute interviews and students were invited to lend their practice assessment booklets so that copies of the ISP assessments could be taken. Seven PEFs, four ECs, 15 mentors and 20 students contributed to the study (see table). Most participants were from the Adult field of nursing. The practice assessment booklets contained examples of ISP use and comments by around 100 mentors *[46].

#### Explanation

Specific kinds of information are required for realist evaluations – some of which comes from respondents or key informants. Data are used to develop and refine theory about how, for whom, and in what circumstances programmes generate their outcomes. This implies that any processes used to invite or recruit individuals need to find those who are able to provide information about contexts, mechanisms and/or outcomes, and that the sample needs to be structured appropriately to support, refute or refine the programme theory [23]. Describing the recruitment process enables judgements to be made about whether the process used is likely to recruit individuals who were likely to have the information needed to support, refute or refine the programme theory.

#### Item 13: Data analysis

Describe in detail how data were analysed. This section should include information on the constructs that were identified, the process of analysis, how the programme theory was further developed, supported, refuted and refined, and (where relevant) how analysis changed as the evaluation unfolded.

#### Example

*The initial programme theory to be tested against our data and refined was therefore that if the student has a trusted assessor (external context) and a learning goal approach (internal context), he or she will find that grades (external context) clarify (mechanism) and energise (mechanism) his or her efforts to find strategies to improve (outcome). …*

*The transcripts were therefore examined for CMO configurations:what effect *(*outcome*) *did the feedback with and without grades have *(*contexts*)*?What caused these effects* (*mechanisms*)*? In what internal and external learning environments *(*contexts*) *did these occur*?

*The first two transcripts were analysed by all authors to develop our joint understanding of what constitutes a context, mechanism and outcome in formative WBA* [*workplace-based assessment*]. *A table was produced for each transcript listing the CMO configurations identified, with columns for student comments about feedback with and without grades (the manipulated variable in the context). Subsequent transcripts were coded separately by JL and AH, who compared their analyses. Where interpretation was difficult, one or two of the other researchers also analysed the transcript to reach a consensus.*

*The authors then compared CMO configurations containing cognitive, self-regulatory and other explanations of the effects of feedback with and without grades, seeking evidence to corroborate and refine the initial programme theory. Where it was not corroborated, alternative explanations that referred to different mechanisms that may have been operating in different contexts were sought *[7].

#### Explanation

In a realist evaluation, the analysis process occurs iteratively. Realist evaluation is usually multi-method or mixed-method. The strategies used to analyse the data and integrate them should be explained. How these data are then used to further develop, support, refute and refine programme theory should also be explained [13]. For example, if interviews were used, how were the interviews analysed? If a survey was also conducted, how was the survey analysed? In addition, how were these two sets of data integrated? The data analyses may be sequential or in parallel – i.e. one set of data may be analysed first and then another or they might be analysed at the same time.

Specifically, at the centre of any realist analysis is the application of a realist philosophical ‘lens’ to data. A realist analysis of data seeks to analyse data using realist concepts. Specifically, realism adheres to a generative explanation for causation – i.e. an outcome (O) of interest was generated by relevant mechanism(s) (M) which were triggered by, or could only operate in, context (C). Within or across the data sources the analysis will need to identify recurrent patterns of CMO configurations [47].

During analysis, the data gathered is used to iteratively develop and refine any initial programme theory (or theories) into one or more realist programme theories for the whole programme. This purpose has implications for the type of data that needs to be gathered – i.e. the data that needs to be gathered must be capable of being used for further programme theory development. The analysis process requires (or results in) inferences about whether a data item is functioning as a context, mechanism or outcome in the particular analysis, but also about the relationships between the contexts, mechanisms and outcomes. In other words the data gathered needs to contain information that [20, 48]:

1. May be used to develop, support, refute or refine the assignment of the conceptual label of C, M or O within a CMO configuration – i.e. in this aspect of the analysis, this item of data is functioning as context within this CMO configuration.
2. Enables evaluators to make inferences about the relationship of contexts, mechanisms and outcomes within a particular CMO configuration.
3. Enables inferences to be made about (and later support, refute or refine) the relationships across CMO configurations – i.e. the location and interactions between CMO configurations within a programme theory.

Ideally, a description should be provided if the data analysis processes evolved as the evaluation took shape.

### Results section

#### Item 14: Details of participants

Report (if applicable) who took part in the evaluation, the details of the data they provided and how the data was used to develop, support, refute or refine programme theory.

#### Example

*We conducted a total of 23 interviews with members of the DHMT [District Health Management Team] (8), district hospital management (4), and sub-district management (7); 4 managers were lost to staff transfers (2 from the DHMT and 2 at the sub-district level). At the regional level, we interviewed 3 out of 4 members of the LDP facilitation team, and one development partner supporting the LDP [Leadership Development Programme]; 17 respondents were women and 6 were men; 3 respondents were in their current posting less than 1 year, 13 between 1–3 years, and 7 between 3–5 years. More than half the respondents (12) had no prior formalised management training *[4].

#### Explanation

One important source of data in a realist evaluation comes from participants (e.g. clients, patients, service providers, policymakers and so on), who may have provided data that were used to inform any initial programme theory or to further develop, support, refute or refine it later in the evaluation.

Item 12 above covers the planned recruitment process and sampling strategy. This item asks evaluators to report the results of recruitment process and sampling strategy – who took part in the evaluation, what data did they provide and how were these data used?

Such information should go some way to ensure transparency and enable judgements about the probative value of the data provided. It is important to report details of who (anonymised if necessary) provided what type of data and how it was used.

This information can be provided here or in Item 12 above.

#### Item 15: Main findings

Present the key findings, linking them to CMO configurations. Show how they were used to further develop, test or refine the programme theory.

#### Example

*By using the ripple effect concept with context-mechanism-outcome configuration (CMOc), trust was at times configured as an aspect of context (i.e. trust/mistrust as a precondition or potential resource), other times as a mechanism (i.e. how stakeholders responded to partnership activities) and was also an outcome (i.e. the result of partnership activities) in dynamically changing partnerships over time. Trust as context, mechanism and outcome in partnerships generated longer term outcomes related to sustainability, spin-off project and systemic transformations. The findings presented below are organized in two sections: (1) Dynamics of trust and (2) Longer-term outcomes including sustainability, spin-off projects and systemic transformations *[38].

#### Explanation

The defining feature of a realist evaluation is that it is explanatory rather than simply descriptive, and that the explanation is consistent with a realist philosophy of science.

A major focus of any realist evaluation is to use the data to support, refute and refine the programme theory – gradually turning it into a realist programme theory. Ideally, in realist evaluations, these processes used to support, refute or refine the programme theory should be explicitly reported.

It is common practice in evaluation reports to align findings against the specific questions for the evaluation. The specific aspects of programme theory that are relevant to the particular question should be identified, the findings for those aspects of programme theory provided, and modifications to those aspects of programme theory made explicit.

The findings in a realist evaluation necessarily include inferences about the links between context, mechanism and outcome and the explanation that accounts for these links. The explanation may draw on formal theories and/or programme theory, or may simply comprise inferences drawn by the evaluators on the basis of the data available. It is important that, where inferences are made, this is clearly articulated. It is also important to include as much detailed data as possible to show how these inferences were arrived at. These data provided may (for example) support inferences about a factor operating as a context within a particular CMO configuration. The theories developed within a realist evaluation often have to be built up from multiple inferences made on data collected from different sources. Providing the details of how and why these inferences were made may require that (where possible) additional files are provided, either online or at request from the evaluation team.

When reporting CMO configurations, evaluators should clearly label what they have categorised as context, what as mechanism and what as outcome within the configuration.

Outcomes will of course need to be reported. Realist evaluations can provide ‘aggregate’ or ‘average’ outcomes. However, a defining feature of a realist evaluation is that outcomes will be disaggregated (for whom, in what contexts) and the differences explained. That is, findings aligned against the realist programme theory are used to explain how and why patterns of outcomes occur for different groups or in different contexts. In other words, the explanation should include a description and explanation of the behaviour of key mechanisms under different contexts in generating outcomes [1, 20].

Transparency of the evaluation processes can be demonstrated, for example, by including such things as extracts from evaluators’ notes, a detailed worked example, verbatim quotes from primary sources, or an exploration of what initially appeared to be disconfirming data (i.e. findings which appeared to refute the programme theory but which, on closer analysis, could be explained by other contextual influences).

Multiple sources of data might be needed to support an evaluative conclusion. It is sometimes appropriate to build the argument for a conclusion as an unfolding narrative in which successive data sources increase the strength of the inferences made and the conclusions drawn.

Where relevant, disagreements or challenges faced by the evaluators in making any inferences should be reported here.

### Discussion section

#### Item 16: Summary of findings

Summarise the main findings with attention to the evaluation questions, purpose of the evaluation, programme theory and intended audience.

#### Example

*The Family by Family Program is young and there is, as yet, relatively little outcomes data available. The findings should, therefore, be treated as tentative and open to revision as further data becomes available. Nevertheless, the outcomes to date appear very positive. The model appears to engage families in genuine need of support, including those who may be considered ‘difficult’ in traditional services and including those with child protection concerns. It appears to enable change and to enable different kinds of families to achieve different kinds of outcomes. It also appears to enable families to start with immediate goals and move on to address more fundamental concerns. The changes that families make appear to generate positive outcomes for both adults and children, the latter including some that are potentially very significant for longer term child development outcomes *[49].

#### Explanation

This section should be succinct and balanced. Specifically for realist evaluations, it should summarise and explain the main findings and their relationships to the purpose of the evaluation, questions and the ‘final’ refined realist programme theory. It should also highlight the strength of evidence [50] for the main conclusions. This should be done with careful attention to the needs of the main users of the evaluation. Some evaluators may choose to combine the summary and conclusions into one section.

#### Item 17: Strengths, limitations and future directions

Discuss both the strengths of the evaluation and its limitations. These should include (but need not be limited to): (1) consideration of all the steps in the evaluation processes and (2) comment on the adequacy, trustworthiness and value of the explanatory insights which emerged.

In many evaluations, there will be an expectation to provide guidance on future directions for the programme, policy or initiative, its implementation and/or design. The particular implications arising from the realist nature of the findings should be reflected in these discussions.

#### Example

*Another is regarding the rare disagreement to what was proposed in the initial model as well as participants infrequently stating what about the AADWP [Aboriginal Alcohol Drug Worker Program] does not work for clients. The fact that participants rarely disagreed with our proposed model may be due to acquiescence to the interviewer, as it is often easier to agree than to disagree. As well, because clients had difficulty generating ways in which the program was not helpful, this may speak to a potential sampling bias, as those clients that have had positive experiences in the program may have been more likely to be recruited and participate in the interviews. This potential issue was addressed by conducting an exploratory phase (Theory Development Phase) and then a confirmatory phase (Evaluation Phase) to try to ensure the most accurate responses were retrieved from a wider variety of participants *[28].

#### Explanation

The strengths and limitations in relation to realist methodology, its application and utility and analysis should be discussed. Any evaluation may be constrained by time and resources, by the skill mix and collective experience of the evaluators, and/or by anticipated or unanticipated challenges in gathering the data or the data itself. For realist evaluations, there may be particular challenges collecting information about mechanisms (which cannot usually be directly observed), or challenges evidencing the relationships between context, mechanism and outcome [19, 20, 22]. Both general and realist-specific limitations should be made explicit so that readers can interpret the findings in light of them. Strengths (e.g. being able to build on emergent findings by iterating the evaluation design) or limitations imposed by any modifications made to the evaluation processes should also be reported and described.

A discussion about strengths and limitations may need to be provided earlier in some evaluation reports (e.g. as part of the methods section).

Realist evaluations are intended to inform policy or programme design and/or to enable refinements to their design. Specific implications for the policy or programme should follow from the nature of realist findings (e.g. for whom, in what contexts, and how). These may be reported here or in Item 19.

#### Item 18: Comparison with existing literature

Where appropriate, compare and contrast the evaluation’s findings with the existing literature on similar programmes, policies or initiatives.

#### Example

*A significant finding in our study is that the main motivating factor is ‘individual’ rather than ‘altruistic’ whereas there is no difference between ‘external’ and ‘internal’ motivation. Previously, the focus has been on internal and external motivators as the key drivers. Knowles et al. (1998), and Merriam and Caffarella (1999), suggested that while adults are responsive to some external motivators (better jobs, promotions, higher salaries, etc.) the most potent motivators are internal such as the desire for increased job satisfaction, self-esteem, quality of life, etc. However, others have argued that to construe motivation as a simple internal or external phenomenon is to deny the very complexity of the human mind (Brissette and Howes 2010; Misch 2002). Our view is that motivation is multifaceted, multidimensional, mutually interactive, and dynamic concept. Thus a person can move between different types of motivation depending on the situation *[51].

#### Explanation

Comparing and contrasting the findings from an evaluation with the existing literature may help readers to put the findings into context. For example, this discussion might cover questions such as how does this evaluation design compare to others (e.g. were they theory-driven?)? What does this evaluation add, and to which body of work does it add? Has this evaluation reached the same or different conclusion to previous evaluations? Has it increased our understanding of a topic previously identified as important by leaders in the field?

Referring back to previous literature can be of great value in realist evaluations. Realist evaluations develop and refine realist programme theory (or theories) to explain observed outcome patterns. The focus on how mechanisms work (or do not) in different contexts potentially enables cumulative knowledge to be developed around families of policies and programmes or across initiatives in different sectors that rely on the same underlying mechanisms [37]. Consequently, reporting for this item should focus on comparing and contrasting the behaviour of key mechanisms under different contexts.

Not all evaluations will be required (or able) to report on this item, although peer-reviewed academic articles will usually be expected to address it.

#### Item 19: Conclusion and recommendations

List the main conclusions that are justified by the analyses of the data. If appropriate, offer recommendations consistent with a realist approach.

#### Example

*Our realist analysis* (Figure 1) *suggests that workforce development efforts for complex, inter-organizational change are likely to meet with greater success when the following contextual features are found*:

- *There is an adequate pool of appropriately skilled and qualified individuals either already working in the organization or available to be recruited.*
- *Provider organizations have good human resources support and a culture that supports staff development and new roles/role design*.
- *Staff roles and identities are enhanced and extended by proposed role changes, rather than undermined or diminished.*
- *The policy context (both national and local) allows negotiation of local development goals, rather than imposing a standard, inflexible set of requirements.*
- *The skills and responsibilities for achieving modernization goals are embedded throughout the workforce, rather than exclusively tied to designated support posts.*

*In reality, however, the optimum conditions for modernization (the right hand side of* Fig. 1) *are rarely found. Some of the soil will be fertile, while other key preconditions will be absent* [52].

#### Explanation

A clear line of reasoning is needed to link the conclusions drawn from the findings with the findings themselves, as presented in the results section. If the evaluation is small or preliminary, or if the strength of evidence behind the inferences is weak, firm implications for practice and policy may be inappropriate. Some evaluators may prefer to present their conclusions alongside their data (i.e. in ‘Item 15: Main findings’).

If recommendations are given, these should be consistent with a realist approach. In particular, if recommendations are based on programme outcome(s), the recommendations themselves should take account of context. For example, if an evaluation found that a programme worked for some people or in some contexts (as would be expected in a realist evaluation), it would be inappropriate to recommend that it be run everywhere for everyone [53]. Similarly, recommendations for programme improvement should be consistent with findings about how the programme has been found to work (or not) – for example, to support the features of implementation that fire ‘positive’ mechanisms in particular contexts, or to redress features that prevent intended mechanisms from firing.

#### Item 20: Funding and conflict of interest

State the funding source (if any) for the evaluation, the role played by the funder (if any) and any conflicts of interests of the evaluators.

#### Example

*The authors thank and acknowledge the funding provided for the Griffith Youth Forensic Service—Neighbourhoods Project by the Department of the Prime Minister and Cabinet. The views expressed are the responsibility of the authors and do not necessarily reflect the views of the Commonwealth Government *[54].

#### Explanation

The source of funding for an evaluation and/or personal conflicts of interests may influence the evaluation questions, methods, data analysis, conclusions and/or recommendations. No evaluation is a ‘view from nowhere’, and readers will be better able to interpret the evaluation if they know why it was done and for which commissioner.

If an evaluation is published, the process for reporting funding and conflicts of interest as set out by the publisher should be followed.

## Discussion

These reporting standards for realist evaluation have been developed by drawing together a range of sources – namely, existing published evaluations, methodological papers, a Delphi panel with feedback comments, discussion and further observations from a mailing list, training sessions and workshops. Our hope is that these standards will lead to greater transparency, consistency and rigour of reporting and, thereby, make realist evaluation reports more accessible, usable and helpful to different stakeholders.

These reporting standards are not a detailed guide on how to undertake a realist evaluation. There are existing resources, published (see Background) and in preparation, that are better suited for this purpose. These standards have been developed to assist the quality of reporting of realist evaluations and the work of publishers, editors and reviewers.

Realist evaluations are used for a broad range of topics and questions by a wide range of evaluators from different backgrounds. When undertaking a realist evaluation, evaluators frequently have to make judgements and inferences. As such, it is impossible to be prescriptive about what exactly must be done in a realist evaluation. The guiding principle is that transparency is important, as this will help readers to decide for themselves if the arguments for the judgements and inferences made were coherent, trustworthy and/or plausible, both for the chosen topic and from a methodological perspective. Within any realist evaluation report, we strongly encourage authors to provide details on what was done, why and how – in particular with respect to the analytic processes used. These standards are intended to supplement rather than replace the exercise of judgement by evaluators, editors, readers and users of realist evaluations. Within each item we have indicated where judgement needs to be exercised.

The explanatory and theory-driven focus of realist evaluation means that detailed data often needs to be reported in order to provide enough support for inferences and/or judgments made. In some cases, the word count and presentational format limitations required by commissioners of evaluations and/or journals may not enable evaluators to fully explain aspects of their work such as how judgments were made or inferences arrived at. Ideally, alternative ways of providing the necessary details should be found such as by online appendices or additional files available from authors on request.

In developing these reporting standards, we were acutely aware that realist evaluations are undertaken by a range of evaluators from different backgrounds on diverse topics. We have therefore tried to produce reporting standards that are not discipline specific but suitable for use by all. To achieve this end, we deliberately recruited panel members from different disciplinary backgrounds, crafted the wording in each item in such a way as to be accessible to evaluators from diverse backgrounds and created flexibility in what needs to be reported and in what order. However, we have not had the opportunity to evaluate the usefulness and impact of these reporting standards. This is an avenue of work which we believe is worth pursuing in the future.

## Conclusions

These reporting standards for realist evaluations have been developed by drawing on a range of sources. We hope that these standards will lead to greater consistency and rigour of reporting and make realist evaluation reports more accessible, usable and helpful to different stakeholders. Realist evaluation is a relatively new approach to evaluation and with increasing use and methodological development changes are likely to be needed to these reporting standards. We hope to continue improving these reporting standards through our email list (www.jiscmail.ac.uk/RAMESES) [21], wider networks, and discussions with evaluators, researchers and those who commission, sponsor, publish and use realist evaluations.
